# The FMRFamide-Like Peptide Family in Nematodes

**DOI:** 10.3389/fendo.2014.00090

**Published:** 2014-06-16

**Authors:** Katleen Peymen, Jan Watteyne, Lotte Frooninckx, Liliane Schoofs, Isabel Beets

**Affiliations:** ^1^Functional Genomics and Proteomics Group, Department of Biology, KU Leuven, Leuven, Belgium

**Keywords:** FMRFamide-like peptides, nematodes, *C. elegans*, neuropeptide, G protein-coupled receptor, feeding behavior, reproduction

## Abstract

In the three decades since the FMRFamide peptide was isolated from the mollusk *Macrocallista nimbosa*, structurally similar peptides sharing a C-terminal RFamide motif have been identified across the animal kingdom. FMRFamide-like peptides (FLPs) represent the largest known family of neuropeptides in invertebrates. In the phylum Nematoda, at least 32 *flp-*genes are classified, making the FLP system of nematodes unusually complex. The diversity of the nematode FLP complement is most extensively mapped in *Caenorhabditis elegans*, where over 70 FLPs have been predicted. FLPs have shown to be expressed in the majority of the 302 *C. elegans* neurons including interneurons, sensory neurons, and motor neurons. The vast expression of FLPs is reflected in the broad functional repertoire of nematode FLP signaling, including neuroendocrine and neuromodulatory effects on locomotory activity, reproduction, feeding, and behavior. In contrast to the many identified nematode FLPs, only few peptides have been assigned a receptor and there is the need to clarify the pathway components and working mechanisms of the FLP signaling network. Here, we review the diversity, distribution, and functions of FLPs in nematodes.

## Introduction

FMRFamide-like peptides (FLPs) are the largest and most diverse family of neuropeptides known (1, 2). Since the identification of the founder sequence FMRFamide from the clam *Macrocallista nimbosa* (3), structurally similar peptides have shown to be present in animals of all major phyla (4, 5). Sequence variants of the authentic tetrapeptide have been mainly identified in lophotrochozoans (6). In most phyla and especially in nematodes, however, a diverse repertoire of extended peptides sharing the C-terminal RFamide motif is found (5, 6). Though, they are thought to have a common eumetazoan origin, the relatedness of subfamilies of FLPs remains unclear because of the large sequence diversity (7, 8). Some peptides show high sequence similarity to FMRFamide suggesting homology to the tetrapeptide, and are therefore often referred to as FMRFamide-related peptides (FaRPs). FaRPs are broadly defined as peptides containing the C-terminal sequence X_1_ X_2_ RFamide, with X_1_ generally representing an aromatic amino acid, whereas X_2_ denotes a hydrophobic residue (6, 7). As many described RFamides differ from the tetrapeptide core for which evolutionary relationships are difficult to determine, the more general term FLP will be used here to address all peptides with a C-terminal RFamide sequence.

FMRFamide-like peptides are intimately involved in a broad pattern of biological processes as diverse as feeding, cardiovascular function, and water homeostasis (4–6, 9). Despite the large sequence diversity, typified by more than 70 family members in the nematode *Caenorhabditis elegans*, several functions of FLPs in the control of energy balance, feeding behavior, reproduction, and neuromodulation emerge consistently throughout evolution (10, 11). Biochemical and genetic studies, exploiting mainly *C. elegans*, have provided insight into the FLP-coordinated regulation of these processes in nematodes. The central role of FLPs in nematode biology including reproduction and locomotory activity has also boosted research on FLP signaling as a target for parasite control in pathogenic nematodes (12–14). However, a lack of data on functional nematode FLP-receptor couples slows down the progress in understanding and exploiting the FLP signaling system. Here, we focus on the evolutionary aspect of FLPs, discussing both the sequence conservation and diversity in the phylum Nematoda, and review our knowledge of conserved FLP signaling functions in nematodes.

## FLP Repertoire of Nematodes

Initial attempts to identify FLPs relied on molecular cloning of *flp-*genes (15, 16), and biochemical characterization of immunoreactive peptide fractions by Edman degradation or gas-phase sequencing [reviewed by Maule et al. (17) and Day and Maule (18)]. The first nematode FLP, named AF1 (KNEFIRFa), was biochemically isolated in this way from the parasite *Ascaris suum* (19). The completion of the *C. elegans* genome sequence revealed a large diversity in the nematode FLP system, boosting the prediction of neuropeptides through *in silico* data-mining (20–23). To date, at least 31 *flp* precursor genes are predicted in *C. elegans* that give rise to around 70 distinct FLPs [Table 1; Ref. (24)]. Expression has been confirmed for the majority of these peptides (Table 1), mainly by peptidomic strategies enabling a comprehensive analysis of the whole peptide content of organisms (25–27). In addition, these approaches allow determining the presence of posttranslational modifications and the exact processing into bioactive peptides, which may be difficult to accurately predict when multiple or non-conventional cleavage sites are present (25). Peptidomic techniques have also been successfully adopted for characterizing and localizing FLPs in other nematodes, mostly in *A. suum* (28–32).

**Table 1:** **Neuropeptide genes encoding FLPs in nematodes**.

*flp* gene^a^	Species^b^	(C-terminal) peptide consensus sequence^c^	*C. elegans* FLPs^d^	*C. elegans flp* expression^e^	*C. elegans* receptor interaction (EC_50_ range)^f^	Reference
*flp-1*	*A. caninum, A. ceylanicum, A. suum, B. malayi, B. xylophilus, C. elegans, C. vulgaris, D. immitis, G. pallida, G. rostochiensis, H. concortus, H. schachtii, L. loa, M. arenaria, M. chitwoodi, M. hapla, M. incognita, M. javanica, M. paranaensis, N. brasiliensis, N. americanus, O. onchengi, O. volvulus, P. redivivus, P. trichosuri, S. ratti, S. stercoralis, T. muris, T. spiralis, W. bancrofti*	-[P/N/Q/A/] [N/T/D/S/K][F/Y]LRFa	**SADPNFLRFa**, **SQPNFLRFa**, **ASGDPNFLRFa**, **SDPNFLRFa**, **AAADPNFLRFa**, (K)PNFLRFa, AGSDPNGLRFa, *(K)PNFMRYa	AIA, AIY, AVA, AVE, AVK, RIG, RMG, M5	NPR-22 (100 nM), NPR-4 (~0.4–9 μM), NPR-11 (~1–8 μM)	(13, 15, 21, 23 –26, 33 –44)
*flp-2*	*A. caninum, A. suum, B. xylophilus, C. elegans, G. pallida, H. concortus, M. chitwoodi, M. hapla, M. incognita, M. javanica, N. americanus, N. brasiliensis, O. ostertagi, S. ratti*	[L/F/V/S/Q][P/R/M][G/R]EP[I/L]RFa	LRGEPIRFa, **SPREPIRFa**	AIA, RID, PVW, I5, MC (ASI, M4, head muscles, an extra pair of cells in the head	FRPR-18 (~50 nM)	(13, 14, 21, 23, 24, 43, 45)
*flp-3*	*A. suum, B. malayi, B. xylophilus, C. elegans, D. immitis, G. pallida, H. glycines, L. loa, M. arenaria, M. chitwoodi, M. hapla, M. incognita, O. volvulus, O. onchengi, S. ratti, W. bancrofti*	-[S/A/E/T/N][P/L][L/F/P]GTMRFa	SPLGTMRFa, **TPLGTMRFa**, **SAEPFGTMRFa**, **NPENDTPFGTMRFa**, **ASEDALFGTMRFa**, EDGNAPFGTMRFa, **EAEEPLGTMRFa**, **SADDSAPFGTMRFa**, NPLGTMRFa	IL1, PQR, SP, CP9	NPR-10 (~60–300 nM), NPR-4 (≥10 μM)	(13, 21, 23 –25, 27, 34, 40, 43, 44, 46, 47)
*flp-4*	*A. caninum, A. ceylanicum, A. suum, B. malayi, B. xylophilus, C. elegans, D. immitis, H. glycines, N. brasiliensis, O. ochengi, O. volvulus, W. bancrofti*	-[A/T/G][Q/N/S/K][P/S][T/S]FIRFa	PTFIRFa, ASPSFIRFa	ADL, ASEL, AVM, AWC, FLP, PHA, PHB, PVD, I5, I6, NSM	NPR-4 (~5–80 nM)	(13, 21, 23, 24, 32, 40, 43, 46)
*flp-5*	*A. caninum, A. suum, B. xylophilus, C. elegans, G. pallida, G. rostochiensis, H. concortus, H. glycines, M. arenaria, M. hapla, M. javanica, M. incognita, N. brasiliensis, N. americanus, P. penetrans, S. ratti*	-[G/A/N/K][A/Q/P]KFIRFa	APKFIRFa, AGAKFIRFa, **GAKFIRFa**	ASE, PVT, RMG, I4, M4, pharyngeal muscle, amphidial neuron (PB, I2), rays 1,5,7, HOB, P8	NPR-11 (~1–8 μM)	(13, 21, 23–25, 34, 40, 43, 44, 46)
*flp-6*	*A. caninum, A. ceylanicum, A. suum, B. malayi, B. xylophilus, C. elegans, D. immitis, G. pallida, G. rostochiensis, H. concortus, H. glycines, L. loa, M. chitwoodi, M. hapla, M. incognita, M. paranaensis, N. brasiliensis, N. americanus, O. ochengi, O. ostertagi, O. volvulus, P. redivivus, S. ratti, S. stercoralis, T. circumcincta, W. bancrofti*	KS[A/S]YMRFa	**KSAYMRFa** (6x), *pQQDSEVEREMM	ASE, AFD, ADF, ASG, PVT, I1 (one or two pairs of head cells), rays 2, 5, 6, 7		(13, 21, 23 –25, 31, 34, 43, 44, 46, 48, 49)
*flp-7*	*A. caninum, A. suum, B. xylophilus, C. elegans, G. pallida, G. rostochiensis, H. concortus, H. glycines, M. hapla, M. incognita, M. javanica, N. brasiliensis, O. ostertagi, S. ratti, S. stercoralis*	[A/T/S]P[F/L/M/I][D/Q/A/E]R[S/A/T], [S/A/T/K][M/L/I][A/V/I]RFa	**TPMQRSSMVRFa** (2x), **SPMQRSSMVRFa** (3x), SPMERSAMVRFa, SPMDRSKMVRFa	ALA, AVG, PHB, PDA, PVW, RIC, SAA (RMDV/SMDV, PHA)	NPR-22 (0.025–5 μM), FRPR-3 (>1 μM)	(13, 21, 24, 26, 34, 35, 43, 44, 46, 50)
*flp-8*	*A. ceylanicum, A. suum, B. malayi, B. xylophilus, C. elegans, D. immitis, H. concortus, L. loa, N. americanus, N. brasiliensis, O. ochengi, O. volvulus, S. ratti, T. muris, T. spiralis, W. bancrofti, X. index*	KNEF[I/V]RFa	**KNEFIRFa** (3x)	AUA, PVM, URX (RMG, ADA, an extra pair of cells in the head), CP9		(13, 19, 21, 23, 24, 29, 34, 43, 46, 51)
*flp-9*	*A. caninum, A. ceylanicum, C. elegans, H. concortus, N. americanus, N. brasiliensis, O. ostertagi*	KPSFVRFa	**KPSFVRFa**		NPR-22 (5 μM)	(13, 21, 24–26, 35, 52)
*flp-10*	*A. ceylanicum, C. elegans, X. index*	-[A/T/M][R/A][S/G][G/S/K]Y[I/L]RFa	QPKARSGYIRFa	AIM, ASI, AUA, BAG, BDU, DVB, PQR, PVR, URX, vulD	EGL-6 (11 nM)	(13, 21, 23, 53)
*flp-11*	*A. suum, A. caninum, A. ceylanicum, B. malayi, B. xylophilus, C. elegans, D. immitis, G. pallida, G. rostochiensis, H. concortus, H. glycines, L. loa, M. hapla, M. incognita, M. paranaensis, N. americanus, N. brasiliensis, O. ochengi, O. ostertagi, O. volvulus, P. penetrans, R. similis, S. ratti, S. stercoralis, T. circumcincta, W. bancrofti*	-M/I/G/A/S][R/A][N/P][A/S/Q/E][P/L], VRFa	**AMRNALVRFa**, **ASGGMRNALVRFa**, **NGAPQPFVRFa**, *SPLDEEDFAPESPLQa	AUA, BAG, VD, DA, DD, DVB, LUA, PHC, PVC, SAB, URX, uvl, head muscle (socket cells), ray 4	NPR-22 (0.75–2.5 μM), FRPR-3 (~1 μM), NPR-4 (≥10 μM)	(13, 21, 23 –26, 28, 29, 35, 40, 43, 46, 50, 54)
*flp-12*	*A. caninum, A. suum, B. malayi, B. xylophilus, C. elegans, D. immitis, G. pallida, G. rostochiensis, H. concortus, H. glycines, L. loa, M. arenaria, M. chitwoodi, M. hapla, M. incognita, M. javanica, M. minor, M. paranaensis, N. americanus, N. brasiliensis, O. ochengi, O. volvulus, S. ratti, W. bancrofti*	(K)[R/K/N]NKFEFIRFa	RNKFEFIRFa	AVA,AVJ, AVH, BAG, PDA, PVR, SAA, SDQ, SMB (BDU), rays 1, 4, 5, 7, CP9		(13, 21, 23, 24, 29, 37 –39, 43, 44, 51, 55)
*flp-13*	*A. caninum, A. ceylanicum, A. suum, B. xylophilus, C. elegans, D. immitis, G. pallida, G. rostochiensis, H. concortus, H. glycines, L. loa, M. chitwoodi, M. hapla, M. incognita, M. javanica, N. americanus, N. brasiliensis, O. ochengi, O. ostertagi, O. volvulus, P. penetrans, P.pacificus, S. ratti, S. stercoralis, W. bancrofti*	-P[F/L/I][I/L/M/V]RFa	**AMDSPFIRFa**, **AADGAPFIRFa**, **APEASPFIRFa** (2x), AADGAPLIRFa, **ASPSAPFIRFa**, **SPSAVPIRFa**, **SAAAPLIRFa**, ASSAPFIRFa	ASE, ASG, ASK, BAG, DD, I5, M3, M5 (an extra pair of cells in the head), VSP	NPR-22 (2.5–5 μM)	(21, 23–26, 31, 35, 43, 44, 46, 51, 56, 57)
*flp-14*	*A. caninum, A. ceylanicum, A. suum, B. malayi, B. xylophilus, C. elegans, D. immitis, G. pallida, G. rostochiensis, H. concortus, L. loa, M. arenaria, M. chitwoodi, M. hapla, M. incognita, M. javanica, M. paranaensis, N. americanus, N. brasiliensis, O. ochengi, O. volvulus, P. redivivus, P. trichosuri, P. penetrans, P.penetrans, R. similis, S. ratti, S. stercoralis, T. circumcincta, T. muris, T. spiralis, W. bancrofti*	KH[E/D][Y/F][L/V/I]RFa	**KHEYLRFa** (4x)		NPR-4 (≥ 10 μM), NPR-11 (~1 – 8 μM)	(13, 22, 24, 26, 29, 31, 34, 37 –40, 43, 44, 51, 55, 58, 59)
*flp-15*	*A. ceylanicum, A. suum, C. elegans, H. concortus, N. americanus, N. braziliensis, O. ostertagi, T. circumcincta*	[R/D/G/A][G/V]P[T/S/Q]GPLRFa	**GGPQGPLRFa**, **RGPSGPLRFa**	PHA, I2, socket/sheath cells (pharyngeal muscle, several cells in the head)	NPR-3 (~100–600 nM), NPR-4 (≥10 μM)	(13, 22 –24, 35, 40, 46, 60)
*flp-16*	*A. caninum, A. ceylanicum, A. suum, B. malayi, B. xylophilus, C. elegans, D. immitis, G. pallida, G. rostochiensis, H. concortus, H. glycines, L. loa, M. hapla, M. incognita, N. americanus, N. brasiliensis, O. ochengi, O. volvulus, O. ostertagi, P. trichosuri, P. penetrans, P. vulnus, R. similis, S. ratti, W. bancrofti*	[A/G]QTFVRFa	**AQTFVRFa** (2x), **GQTFVRFa**			(13, 24, 43, 44, 46)
*flp-17*	*A. caninum, A. suum, B. xylophilus, C. elegans, H. contortus, N. americanus, N. brasiliensis, O. ostertagi, S. ratti, S. stercoralis, X. index*	KS [A/S/Q][F/Y/L][V/I]RFa	KSAFVRFa (2x), KSQYIRFa	BAG, M5 (an extra pair of cells in the head), rays 1, 5, 7	EGL-6 (1–28 nM)	(13, 22 –24, 32, 43, 46, 53)
*flp-18*	*A. caninum, A. ceylanicum, A. suum, B. xylophilus, C. elegans, D. immitis, G. pallida, G. rostochiensis, H. concortus, L. loa, M. chitwoodi, M. hapla, M. incognita, M. javanica, N. americanus, N. brasiliensis, O. ochengi, O. ostertagi, O. volvulus, P. pacificus, S. ratti, S. stercoralis, T. muris, T. spiralis, W. bancrofti*	-[P/Q/A][G/Q/D/A], [V/M/F/L][V/M/F/L]RFa	(DFD)GAMPGVLRFa, EMPGVLRFa, (SYFDEKK)SVPGVLRFa (3x), EIPGVLRFa, SEVPGVLRFa, DVPGVLRFa	AVA, AIY, RIG, RIM, M2 (M3, two extra pairs of cells in the head), rays 2, 6	NPR-4 (~5–80 nM), NPR-10 (~60 nM–4.6 μM), NPR-1 [(−32.2)–(−6.8)]**, NPR-5a (~20–70 μM), NPR-5b (~30–800 nM), NPR-11 (~80 nM–8 μM)	(13, 16, 24–26, 29, 37, 39, 40, 43, 44, 46, 61, 62)
*flp-19*	*A. caninum, A. suum, B. malayi, B. xylophilus, C. elegans, D.immitis, G. pallida, H. concortus, H. glycines, L. loa, M. hapla, M. incognita, N. americanus, N. brasiliensis, O. ochengi, O. volvulus, P. penetrans, S. ratti, T. circumcincta, W. bancrofti*	-W[A/S][N/S/T][Q/K/S][V/L]RFa	**WANQVRFa**, **ASWASSVRFa**	AIN, AWA, BAG, HSN, URX (an extra pair of cells in the tail), rays 5, 7, 9, CEM		(13, 22 –26, 32, 34, 43, 44, 46)
*flp-20*	*A. suum, A. caninum, B. xylophilus, C. elegans, G. pallida, H. concortus, M. hapla, M. incognita, N. brasiliensis, P. trichosuri, S. ratti*	[A/V]MMRFa	AMMRFa (2x)	ALM, ASEL, AVM, LUA, PLM, PVC, PVM, PVR, RIB, AIB (PVT)		(13, 22 –24, 43, 44)
*flp-21*	*A. caninum, A. suum, B. malayi, B. xylophilus, C. elegans, D. immitis, G. pallida, H. concortus, L. loa, M. hapla, M. incognita, N. americanus, N. brasiliensis, O. ochengi, O. ostertagi, O. volvulus, P. penetrans, P. pacificus, R. similis, S. ratti, S. stercoralis, T. circumcincta, W. bancrofti*	-[G/A/S/L][L/A]GPRPLRFa	GLGPRPLRFa	ADL, ASI, ASEASH, ASJ, ASK, FLP, URA, MC, M4, M2, SP, DVF, P6, P7, P9	NPR-1 (~2.5–100 nM), NPR-11 (~1–10 nM), NPR-5a (~0.6–5 μM), NPR-5b (~200–1500 nM)	(13, 24, 40, 43, 44, 47, 61–63)
*flp-22*	*A. caninum, A. ceylanicum, A. suum, B. malayi, B. xylophilus, C. elegans, D. immitis, G. pallida, G. rostochiensis, H. concortus, H. glycines, L. loa, M. hapla, M. incognita, N. brasiliensis, O. ochengi, O. ostertagi, O. volvulus, P. trichosuri, P. penetrans, P. pacificus, R. similis, S. ratti, S. stercoralis, T. circumcincta, W. bancrofti*	-[P/E/A/T/S][P/Q/G/E/N/S][S/G/V/A], KWMRFa	**SPSAKWMRFa** (3x)	AIM, ASG, AVA, AVG, AVL, CEP, PVD, PVW, RIC,AIZ, RIV, SMD, URA, uvl, 6 out of 9 CP	NPR-22 (1 μM)	(13, 24–26, 35, 43, 44)
*flp-23*	*B. malayi, C. elegans, D. immitis, L. loa, O. ochengi, O. volvulus, T. circumcincta, W. bancrofti*	-[V/I/T][V/D/K][G/D/F][Q/G/F]QDFLRFa	VVGQQDFLRFa, TKFQDFLRFa			(13, 23, 24, 46)
*flp-24*	*A. caninum, A. ceylanicum, A. suum, B. malayi, C. elegans, D. immitis, H. concortus, L. loa, N. americanus, O. ostertagi, O. ochengi, O. volvulus, S. ratti, W. bancrofti*	VP[S/N][A/P][G/A]DMM[V/I]RFa	**VPSAGDMMVRFa**			(13, 23, 24, 31, 46)
*flp-25*	*A. caninum, A. suum, B. malayi, C. elegans, D. immitis, G. pallida, G. rostochiensis, H. concortus, L. loa, M. chitwoodi, M. hapla, M. incognita, M. javanica, N. americanus, N. brasiliensis, O. ochengi, O. volvulus, S. ratti, S. stercoralis, W. bancrofti*	-[D/A/S/N/T]YD[Y/F][V/I]RFa	DYDFVRFa, **ASYDYIRFa**	ASE		(13, 24, 26, 44, 46, 64)
*flp-26*	*A. caninum, A. ceylanicum, A. suum, C.elegans, N. americanus*	-[G/S][G/E][G/E/P][L/M/I][A/E]F[H/S/N], [P/A][N/D][D/M]L[A/S/T]LRFa	**(E)FNADDLTLRFa**, **GGAGEPLAFSPDMLSLRFa**, *FRLPFQFFGANEDFNSGLT, *NYYESKPY			(13, 24, 26, 46)
*flp-27*	*A. caninum, C. elegans, H. glycines, M. chitwoodi, M. hapla, M. incognita, M. javanica, M. paranaensis, N. americanus, R. similis*	[G/T/S/A][K/L/M]G[G/S]RMRFa	GLGGRMRFa, *pQPIDEERPIFME			(13, 24, 26, 44, 46)
*flp-28*	*A. suum, A. caninum, C. elegans, H. concortus, N. brasiliensis, O. ostertagi, P. penetrans, S. ratti*	-[V/I][L/F]MRFa	VLMRFa, **APNRVLMRFa**			(13, 24, 26)
*flp-31*	*B. xylophilus, G. pallida, M. chitwoodi, M. hapla, M. incognita, P. penetrans*	LYRPRGPPRFa				(13, 24, 44)
*flp-32*	*A. caninum, B. xylophilus, C. elegans, G. pallida, H. concortus, M. hapla, M. incognita, N. brasiliensis, S. ratti*		AMRNSLVRFa			(13, 24, 43, 44, 46)
*flp-33*	*A. suum, A. caninum, B. xylophilus, C. elegans, H. concortus, N. brasiliensis*		**APLEGFEDMSGFLRTIDGIQ**, **KPRFa**			(24, 43, 46, 65)
*flp-34*	*A. suum, A. caninum, B. malayi, B. xylophilus, C. elegans, D. immitis, G. pallida, H. concortus, M. hapla, M. incognita, L. loa, N. brasiliensis, O. onchengi, O. volvulus, W. bancrofti*		ALNRDSLVASLNNAERLRFa, *ADISTFASAINNAGRLRYa			(24, 46)

*^a^The *flp*-coding genes *flp-29* and *flp-30* were recently suggested to represent orthologs of *C. elegans flp-28* and *flp-2*, respectively, and have been accordingly included in this table (24)*.

*^b^Species: *Ascaris suum, Ancylostoma caninum, Ancylostoma ceylanicum, Brugia malayi, Bursaphelenchus xylophilus, Caenorhabditis elegans, Caenorhabditis vulgaris, Dirofilaria immitis, Globodera pallida, Globodera rostochiensis, Haemonchus concortus, Heterodera glycines, Heterodera schachtii, Loa loa, Meloidogyne arenaria, Meloidogyne incognita, Meloidogyne javanica, Meloidogyne hapla, Meloidogyne paranaensis, Necator americanus, Nippostrongylus braziliensis, Onchocerca ochengi, Onchocerca volvulus, Ostertagia ostertagi, Panagrellus redivivus, Parastrongyloides trichosuri, Pratylenchus penetrans, Pristionchus pacificus, Radolphus similis, Strongyloïdes ratti, Strongyloïdes stercoralis, Teladorsagia circumcincta, Trichinella spiralis, Trichuris muris, Wuchereria bancrofti*, and *Xiphinema index**.

*^c^Sequences that start with a hyphen have variable N-terminal extensions*.

*^d^Peptides indicated in bold have been isolated from *C. elegans*. Peptides indicated with an asterisks are non-FLPs encoded by the indicated *flp* gene. The copy number of peptides encoded by the gene is indicated between brackets*.

*^e^Expression patterns were adapted from Ref. (46, 64) and Wormbase (http://www.wormbase.org)*.

*^f^The approximate EC_50_ range for receptor activation is indicated between brackets and includes receptor activation by all peptides encoded by this precursor*.

**Values represent alteration of current in response to neuropeptide application in *Xenopus* assay.

Although the FLP repertoire of nematodes has been best studied in *C. elegans*, evidence emerges on the distribution of FLPs in parasitic and free-living species across the nematode phylum (Table 1). Complete genome sequences have been determined for few phylum members so far, but transcriptome data is available for over 60 nematode species (66, 67). In 2005, McVeigh and co-workers performed a systematic BLAST analysis of EST databases to investigate FLP sequence diversity within the phylum Nematoda identifying more than 500 FLPs across 46 species (13). The FLP complement of other nematodes seems to be generally similar to that of *C. elegans* (13, 30, 44). This finding is confirmed by a very recent study of McCoy and colleagues, who re-investigated the available genome and transcriptome resources for 17 pathogenic nematodes (24). Many nematode FLPs display a high degree of inter-species structural conservation that is independent of their parasitic or free-living lifestyle [Table 1; Ref. (13)], supporting a fundamental role of FLPs in nematode biology. Corroborating this, only one nematode *flp* gene is thought to be parasite-specific; the *flp-31* gene is absent from the *C. elegans* genome, but occurs in several plant parasitic nematodes suggesting a function specific in phytoparasitism (13, 24, 44). Although *flp-31* was previously predicted from *A. suum* (32), this gene is considered to be a sequelog of *C. elegans flp-15* (24). Initially, *flp*-29 and *flp*-30 were also found to be parasite-specific (13), but a recent investigation of their C-terminal motif and genomic location suggests that these genes should be re-designated to respectively *flp*-28 and *flp*-2 (24).

Whereas most FLPs are likely widespread throughout the nematode phylum, variable conservation has been reported for some family members. Highly conserved nematode *flp-*genes include *flp-1, flp-6, flp-11, flp-12, flp-14, flp-16, flp-18, flp-19, flp-21*, and *flp-22*; other genes such as *flp-2* and *flp-10* have shown to be more restricted and structurally diverse (13, 24). Interestingly, parasitic nematodes appear to possess variable proportions of the *C. elegans flp*-gene complement and variation is highest among distinct clades (24). Genome-wide analysis of the parasite *Meloidogyne incognita* showed that its FLP complement is reduced to about 60% compared to that of *C. elegans* (44). Two other nematodes, *Trichuris muris* and *Trichinella spiralis*, were shown to display a dramatically reduced complement of only 13%, whereas *A. suum* possesses 84% of *C. elegans flp*-genes (24) The finding that fewer *flp-*genes are expressed in parasitic nematodes as compared to free-living species has been postulated to be an indication of the more contained repertoire of stimuli these nematodes encounter during their endoparasitic stage (68). Furthermore, more FLPs seem to be present in the animal parasitic datasets compared to plant parasitic nematodes (68). Our view on the diversity of FLPs in nematodes however strongly depends on the available sequence data. In depth analyses of the increasing number of completed genome sequences and transcriptome resources should further expand our understanding of the nematode FLP repertoire in the near future.

Recent studies estimate the presence of 32 distinct *flp-*genes in nematodes (24). Among them are 15 genes that code for N-terminally extended peptides carrying the classical FaRP motif, whereas most others peptides share the restricted RFamide core (Table 1). Although the relatedness of FLPs across metazoans is often unclear, sequences of the neuropeptide F (NPF) family have been identified in several invertebrate groups and predicted in the nematode phylum as well (69). NPF-like peptides are encoded by the *flp-27* precursor that is highly conserved in nematodes, and contains the C-terminal RXRFamide motif characteristic of the invertebrate NPF family (70). The plethora of FLPs in nematodes is high, given the structural simplicity of their nervous system harboring around 300 neurons (2, 5, 6). This diversity of neural messengers is magnified by classical neurotransmitters and a broad range of other neuropeptides of insulin (*ins*) and neuropeptide-like protein (*nlp*) families, of which about 200 peptides are predicted in *C. elegans* (46, 71).

## Localization of FLPs

Immunocytochemical localization of FLPs has been performed in various nematode species, mainly using antibodies raised against synthetic FMRFamide or the RFamide motif (18, 37, 55). These studies suggest that FLPs are widely expressed in the nervous system of all nematodes, supporting a general role for FLP signaling in nematode biology. The broad distribution of immunoreactive neurons, including in motor neurons, fueled the research effort to decipher nematode FLP signaling and its role in neuromotor function, which had already proven to be a successful target for parasite control (12). Although the vast patterns of FLP immunoreactivity are generally similar between nematode species, HPLC-ELISA studies have identified qualitative differences between free-living and plant parasitic nematodes, suggesting that the distinct peptides present in plant nematodes are structurally different (72, 73).

Despite immunocytochemistry being immensely useful to study gross patterns of FLP localization, most of the C-terminally directed antibodies used were incapable of reliably discriminating between structurally related FLPs. Gene-specific *flp* expression has been mainly investigated in *C. elegans*, using reporter transgenes in which LacZ or a fluorescent protein gene is placed under the control of the endogenous promoter region. Li and co-workers applied this molecular approach to map the specific expression patterns of *flp-1* to *flp-23* genes (22, 23). Just over 50% of the total number of neurons were found to express *flp’s*, a wide distribution in stark contrast to earlier immunochemical studies in which only 10% of all *C. elegans* neurons showed FLP reactivity (74). Expression could be detected in all neuronal cell types, including interneurons, sensory neurons, and motor neurons. Six *flp-*genes were also expressed in non-neuronal cells, including in head muscle (*flp-2* and *flp-11*), pharyngeal muscle (*flp-5* and *flp-15*), socket and/or sheath cells (*flp-11* and *flp-15*), vulval cells (*flp-10*), and uterine cells (*flp-11* and *flp-2*). Although the expression of each *flp* gene can be precisely delineated, there is a considerable overlap with many cells expressing more than one *flp* gene (23). Most *flp-*genes are also expressed in multiple neurons suggesting that some FLPs have overlapping functions, unlike others fulfilling unique roles.

*In situ* hybridization (ISH), which uses nucleotide probes complementary to specific gene transcripts, offers an attractive alternative to delineate *flp* gene expression in other nematodes that are less amenable to transgenesis than *C. elegans* (38, 42, 54, 75). Furthermore, as immunochemistry allows the determination of neurite morphology facilitating neuronal identification, several antibodies highly specific to certain *A. suum* FLPs were recently generated (42, 76). Another approach for FLP localization consists of direct mass spectrometric analysis on dissected neuronal tissue without the need of an extensive extraction process (29, 76). Importantly, this technique can identify previously unknown peptides as no prior sequence information is required. Yew et al. (29) subjected individually dissected nerve ganglia from *A. suum* to mass spectrometric analysis, producing a peptidomic map of the individual anterior ganglions. FLP distribution appeared to be much less restricted to specific cells as compared to the gene expression studies in *C. elegans* or *G. pallida* using reporter constructs and ISH, thus supporting the notion that FLP expression differs among nematode species. The authors however also stress the sampling biases inherent in the use of mass spectrometry. In large nematodes such as the foot-long *A. suum*, cell-specific FLP content can also be rapidly determined by precisely dissecting individual cells (31, 42).

Taken together, abovementioned studies paint a picture in which FLPs widely occur in all known nematode neuronal subtypes and even in non-neuronal tissues. Whereas the gross patterns of FLP distribution remain consistent across the phylum, more recent studies indicate that the cellular expression of homologous FLPs can substantially differ between nematode species (2, 42). This is remarkable, given that both the general FLP complement and the basic nervous architecture are conserved, with *C. elegans* even considered as a miniature version of *A. suum* at the level of neuronal morphology (2, 24, 51, 77, 78). Caution is however warranted as dissimilarities could be attributed to experimental differences, with each technique suffering from inherent caveats. Besides the limited specificity of the commonly used antibodies, transgenic reporter constructs may not contain all regulatory sequences necessary to recapitulate endogenous gene expression. Moreover, variability of cellular expression patterns of different gene products has repeatedly been observed in *A. suum*, partially due to genetic differences since the worms are not isogenic (31, 42, 75, 76).

## FLP-Receptors in Nematodes

Most FLPs are known to act through binding of G protein-coupled receptors (GPCRs) (79–81). Although the early work on nematode FLPs primarily focused on peptide identifications, *in vitro* and functional studies have started to address the biology of their receptors and mode of action. In *C. elegans*, sequence similarity or homology to the FLP-receptor family has been postulated for several of the more than 100 peptide GPCR genes predicted in the genome (81, 82). The neuropeptide receptor NPR-1 was previously suggested as a member of the invertebrate NPF receptor (NPFR) family and related neuropeptide Y receptors (NPYRs) in mammals (83). Sequence similarity and phylogenetic clustering suggests additional NPFR/NPYR-like family members are likely to be present in *C. elegans*, as well as representatives related to vertebrate neuropeptide FF receptors, and *Drosophila* myosuppressin and FMRFamide receptors (8, 82, 84). Peptides that functionally activate these GPCRs, with exception of NPR-1, unfortunately remain unknown.

In general, few nematode FLPs have been matched to their receptor(s) and the identification of FLP-receptor couples has only been undertaken in *C. elegans* (2, 81). Activation by FLPs has been reported for 13 *C. elegans* receptors encoded by 10 genes (Table 1), all of which are members of the rhodopsin family of GPCRs [reviewed in Ref. (81)]. Deorphanization, i.e., the identification of receptor ligand(s), is typically done by expressing GPCRs in a heterologous cellular system such as *Xenopus* oocytes, mammalian cells or yeast. Receptor activation can then be detected by monitoring downstream steps in the GPCR signaling pathway including levels of secondary messenger molecules or GTP exchange upon G protein activation (85). When heterologous GPCRs are challenged with a peptide library, multiple FLPs are generally found to activate a single receptor. Peptide motifs essential for receptor activation are often shared by FLPs derived from the same precursor protein. For example, Kubiak and co-workers showed that all peptides from the FLP-15 precursor carrying the highly similar GPXGPLRFamide motif, recognize the neuropeptide receptor NPR-3 (60). Likewise, two structurally similar FLPs processed from the FLP-2 precursor were found to activate *C. elegans* receptors encoded by the *frpr-18* locus (45). By monitoring intracellular calcium levels, Mertens et al. showed that both FLP-2 peptides activate two isoforms of the receptor FRPR-18 though with different potencies. Whereas SPREPIRFamide (FLP-2A) was active with nanomolar half-maximal effective concentrations (EC_50_ values), FRPR-18 receptors were only activated at micromolar concentrations by LRGEPIRFamide (FLP-2B). In contrast, Larsen and co-workers found FLP-2A and FLP-2B to be equipotent on the FRPR-18b isoform using a similar calcium mobilization assay in a different type of cells (14). Receptor pharmacology can thus vary dependent on the heterologous system, which may be due to differences in the available G protein signaling machinery or folding properties that affect the functional expression of a GPCR. Although *in vitro* expression systems may not fully reflect endogenous settings, most ligand-receptor couples identified in *C. elegans* are supported by functional studies on FLPs and their receptors (61, 62, 81). Functional evidence on peptide GPCRs and putative FLP ligands is also emerging in other nematodes (86, 87), which may serve as a lead in the search for FLP-receptors in these species.

In *C. elegans* and likely other nematodes, the FLP signaling network is highly expanded by GPCRs able of binding multiple FLPs that, can even originate from different precursor proteins (Table 1). The neuropeptide receptor NPR-1 was the first FLP-receptor to be deorphanized in *C. elegans*, and shown to recognize both FLP-18 and FLP-21 peptides (61, 63). Interestingly, this GPCR exists in two variants differing by a single amino acid at position 215, NPR-1.215F or NPR-1.215V that is likely implicated in G protein coupling. Substitution of this residue is sufficient for affecting ligand binding and potency resulting in the differential regulation of feeding behavior (61, 63, 83). Both receptor variants are activated by the FLP-21 peptide that is, however, 10-fold more potent in binding NPR-1.215V than NPR-1.215F (61, 63). In addition, Rogers and co-workers found that the NPR-1.215V variant expressed in *Xenopus* oocytes can be activated by peptides from the FLP-18 precursor, albeit with lower potencies than FLP-21 (61). A second study reported by Kubiak et al. did not identify FLP-18 peptides as the ligands of NPR-1.215V (63), but the receptor variant was expressed in mammalian cells and differences in expression system may account for the discrepancy in identified ligands in both studies. Both FLP-21 and FLP-18 peptides have been found to activate other *C. elegans* receptors as well, including NPR-11 and NPR-5 (40, 62, 88). In addition, FLP-18 peptides were also identified as ligands of the receptors NPR-4 and NPR-10 (40, 62). An unusual structure-activity relationship has been suggested for the *C. elegans* receptor FRPR-3 (47, 50). Ligands identified for this GPCR include a FLP-7 (TPMQRSSMVRFamide) and FLP-11 (AMRNALVRFamide) peptide, whereas structurally similar peptides encoded on the same precursor proteins were ineffective at activating the receptor (50). However, it should be noted that EC_50_ values for both peptides reside in the micromolar range (50), and other functional ligands might activate FRPR-3 with higher potency. FLP-7 and FLP-11 peptides were also shown to activate another receptor, NPR-22, together with an array of FLPs including FLP-1, FLP-9, FLP-13, and FLP-22 peptides (35). The potencies of receptor activation varied from the nanomolar to the micromolar range (Table 1). Finally, the EGL-6 receptor found to be involved in *C. elegans* egg-laying has been coupled to its FLP-10 and FLP-17 ligands in two ways, by making use of an *in vitro* assay but also by screening neuropeptide-encoding transgenes for the ability to inhibit egg-laying (53).

Although knowledge has been gathered on the receptor biology of several *C. elegans* FLPs, our view of nematode FLP-receptors is far from complete. Deorphanization of GPCRs has been successful in matching some FLP-receptor couples; however, often a sub-set of the predicted peptide repertoire is tested such that the array of ligands acting on a receptor remains incomplete. FLPs are thought to exert most of their effects through the activation of GPCRs, but some family members are capable of eliciting fast responses by gating ion channels (89–91). This mode of action likely also applies for several nematode FLPs (90–94). The coupling of multiple peptides to a single GPCR and vice versa greatly enhances the complexity of FLP signaling in *C. elegans*. However, the characterization of all functional FLP-receptor couples will be crucial to further expand our understanding of nematode FLP signaling, and will uncover whether promiscuity of FLP-receptors can be generalized in nematodes.

## FLP-Mediated Modulation of Nematode Physiology and Behavior

Despite the apparent simplicity of the nematode nervous system, harboring around 300 cells, a surprisingly rich behavioral repertoire has been described (86, 87, 95, 96). The structural and spatiotemporal gene expression diversity of the nematode FLP system is reflected in the range of FLP-induced physiological responses. The role of FLPs has been extensively described in previous reviews (2, 5, 6); here, we focus on FLP signaling functions emerging consistently throughout evolution to illustrate some of the general principles of FLP signaling gleaned from the study of nematode peptides. Although *C. elegans* has been heavily exploited to investigate the basic biology of FLP signaling, we highlight some pharmacological and behavioral studies performed on related nematodes.

### Nematode FLPs in the control of feeding behavior

Although FLPs display a tremendous diversity in structure and biological activity, their involvement in the regulation of energy balance and feeding behavior has been described in both invertebrate and vertebrate lineages (11). Feeding state is a paramount environmental factor that guides *C. elegans* behavior, with a central role for FLP signaling in for instance the regulation of locomotory activity, foraging and food intake (5, 6, 46).

#### *C. elegans* NPR-1 signaling regulates food-dependent aggregation behavior

The best characterized example of FLP-modulated behavior in *C. elegans* is food-related aggregation. Certain wild-type isolates, including the standard laboratory strain N2, mainly show a “solitary feeding” phenotype in which worms disperse to feed alone. Others have a propensity to aggregate into clumps in areas of high food density, a behavior that is termed “social feeding” (83). This behavioral polymorphism can be attributed to a single amino acid difference in the *npr-1* gene, which encodes a member of the NPYR/NPFR family (83). Worms expressing the partial loss-of-function isoform with a phenylalanine, NPR-1.215F, are social feeders, whereas strains bearing the *npr-1* allele encoding the version with a valine, NPR-1.215V, are solitary. Since chemically generated null mutations of *npr-1* convert the solitary wild-type N2 lab strain into an aggregating one, NPR-1 activity is suggested to repress aggregating behavior (83).

Both loss-of-function and gain-of-function studies confirm that FLP-21 acts as the endogenous NPR-1 ligand required for its activation and consequent suppression of food-dependent aggregation (61). Whereas transgenic overexpression of *flp-21* rescues the social feeding phenotype of NPR-1.215F worms, genomic deletion of *flp-21* further enhances worm clumping. However, loss of *flp-21* only slightly increases aggregation in animals bearing the Val-215 allele, suggesting that another ligand most likely encoded by the *flp-18* gene may functionally substitute for the loss of FLP-21 ligands (61, 95). FLP-21 furthermore does not appear to act in NPR-1 dependent acute ethanol tolerance, once again suggesting that FLP-18 may be a physiological active ligand (97).

The food-dependent aggregation of social *npr-1* mutant worms relies on chemosensory responses in a number of different sensory neurons exposed to the environment and the pseudocoelomic body fluid. Due to their specific localization in *C. elegans*, these cells are able to detect various adverse or stressful conditions (98, 99). Despite expression of *npr-1* in at least 20 neurons (99), the inter/motorneuron RMG seems to be the cellular hub of the NPR-1 mediated feeding behavior (100). Anatomical gap junctions connect RMG to five sensory neurons known to promote aggregation, including the nociceptive ASH and ADL neurons and the URX oxygen sensor (101). In a hub-and-spoke model in which RMG functions as the central hub, RMG is suggested to integrate signals from various sensory neurons to stimulate aggregation using its own chemical synapses. Furthermore, due to the bidirectionality of the gap junctions, RMG in turn modulates the responses of its associated sensory neurons having their own synaptic outputs (100). NPR-1 however inhibits the gap junction driven activation of RMG, either by downregulating the gap junctions or by altering RMG excitability (99, 100).

NPR-1 functions as a modulator of many neurons and behavioral responses, not only in response to food but also to other key environmental parameters of which ambient O_2_ and CO_2_ levels appear to have a major influence. NPR-1 modulates aerotaxis as well as the integration of sensory cues of food availability, internal metabolic state and O_2_ levels (100, 102–106). NPR-1 also alters the sensitivity to environmental repellents (e.g., pheromones, CO_2_), innate immune responses, and tolerance to ethanol (107–113). The fact that *flp-18* and *flp-21* mutants often display more subtle phenotypes in these studies indicates that NPR-1 might have additional ligands. Furthermore, the expression patterns of *flp-18* and *flp-21* have limited overlap and it is not known how the expression of both genes or release of their peptide products is regulated. Despite the wide neuronal expression pattern of *npr-1*, RMG can be pinpointed as the cellular locus of NPR-1 function for a number of behaviors. Besides “social feeding,” *npr-1* expression in RMG acts synergistically with the primary heat sensing machinery to regulate aversive behaviors at high temperature, with *npr-1* or *flp-21* loss-of-function animals showing an increased threshold for heat avoidance (112). Similarly, RMG-specific rescue of *npr-1* restores pheromone avoidance defects in the *npr-1* mutant background (114). Furthermore, NPR-1 and its FLP-18 and FLP-21 ligands are required for locomotion quiescence during lethargus, a quiescent behavioral state occurring before each of the four molts, with increased activity in the RMG circuit promoting locomotion arousal (115). The RMG-hub-and-spoke circuit therefore appears to be a multifunctional sensory circuit integrating various stimuli that heavily depends on FLP neuropeptidergic signaling in order to coordinate behavioral output.

#### Other FLPs modulating food-related behaviors

**Figure 1:**
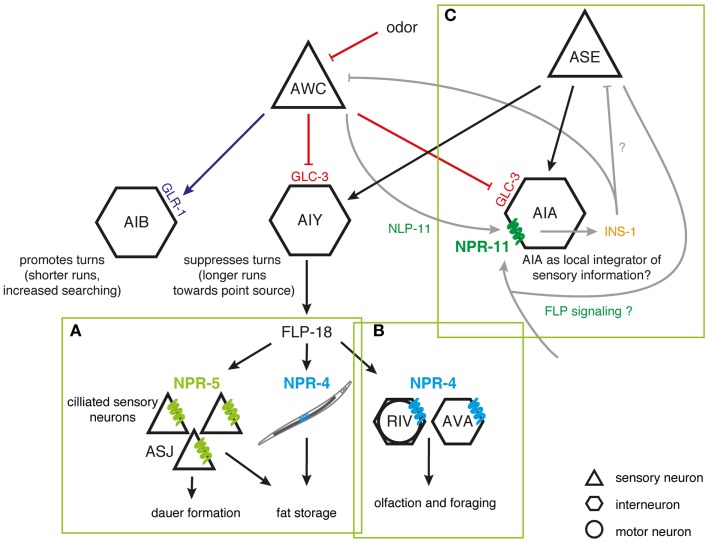
**FLP signaling regulates *C. elegans* foraging and metabolism**. **(A)** FLP-18 peptides are released from AIY in response to sensory cues relaying food availability. By acting on the receptors NPR-4 in the intestine and NPR-5 in ciliated neurons, FLP-18 peptides control fat storage; while activation of NPR-5 in ASJ neurons regulates dauer formation. **(B)** To regulate odor responses and foraging strategy, FLP-18 peptides signal through NPR-4 in AVA and RIV neurons that control reversal frequency and turning bias, respectively. **(C)** Peptidergic feedback modulates sensory responses in *C. elegans*. In response to odor, the AWC olfactory neuron releases NLP-1 neuropeptides, which act on the NPR-11 receptor on AIA to modulate INS-1 peptide secretion. INS-1 subsequently closes the feedback loop by modulating AWC’s responsiveness to sensory stimuli. AIA could act as a local integrator of sensory information, with FLP sensory peptides driving similar neuropeptidergic feedback loops to modulate the responsiveness to sensory stimuli [adapted from Ref. (62, 117–121)].

Besides aggregation, FLPs are implicated in other feeding behaviors such as the regulation of energy balance and metabolism according to perceived food availability (11). In *C. elegans*, loss-of-function of the *flp-18* precursor gene causes defects in chemosensation, foraging, and formation of the arrested dauer developmental stage that is induced by stress conditions (62). In addition, these mutants display increased levels in intestinal fat and reduced aerobic metabolism, strongly suggesting that FLP-18 neuropeptides are involved in fat storage and metabolism (62). FLP-18 peptides activate the neuropeptide receptors NPR-4 and NPR-5, and loss-of-function of these GPCRs recapitulates some of the phenotypic effects observed in *flp-18* mutants (62, 88). Cohen and co-workers found that *npr-4* is expressed in a number of sites including the intestine, whereas NPR-5 is present in several sensory neurons and head, neck, and body wall muscles. FLP-18 signaling through activation of NPR-4 in intestinal muscle was shown to regulate the accumulation of intestinal fat. NPR-5 however modulates the activity of a number of amphid sensory neurons that directly sense environmental cues, of which the chemosensory ASJ neurons are critical in dauer formation (Figure 1A) (116).

Environmental food availability also strongly influences *C. elegans* food-seeking behavior. When feeding on a bacterial lawn, *C. elegans* spends most of its time slowly moving within a restricted area. Upon removal from their food source, animals however initiate an intensive local search behavior characterized by repetitive bursts of reversing and turning in a restricted immediate area. After prolonged food withdrawal (≥15 min off-food), FLP-18 neuropeptides released from the primary interneuron AIY and to a lesser extent from RIG interneurons, activate a switch in behavioral state from this local search to dispersal in which turning events are suppressed (62, 117). FLP-18 peptides were found to act on the neuropeptide receptor NPR-4 in AVA interneurons and RIV motor neurons that regulate reversal frequency and turning bias, respectively (Figure 1B) (117, 122). NPR-4 signaling by FLP-18 peptides may therefore reduce local search behavior by modulating the activity of these neurons (62). Upstream in the circuit, AIY interneurons, which release FLP-18, receive synaptic input from various sensory neurons. As such, they presumably play an integrative role enabling the regulation of locomotory behaviors in response to environmental perception. Among the presynaptic partners is the AWC olfactory neuron pair that is a prominent player in local search behavior (117). Both neurons are stimulated following the removal of an attractive odorant that serves as a cue for food presence (119). Upon odorant removal, the AWC neurons provide glutamatergic input to downstream interneurons that will accordingly reorient locomotory behavior by stimulating local search behavior. Glutamate release was found to hyperpolarize AIY neurons that express FLP-18 and suppress turning, via the glutamate-gated Cl^−^ channel GLC-3. On the other hand, AIB interneurons that promote turning are depolarized by glutamate via the AMPA/kainate-like glutamate receptor GLR-1, resulting in directed chemotaxis behavior along odor gradients (119, 120, 123). Taken together, these observations fit within a model in which sensory detection of food availability can coordinately regulate adequate responses such as foraging behavior and energy metabolism via FLP signaling (Figure 1).

Interestingly, there is evidence for a neuropeptide-mediated sensorimotor feedback loop that dampens the odor-evoked activity of the AWC neurons, hereby limiting local search behavior (120). When odor is sensed, the AWC neurons release buccalin-related NLP-1 peptides, which in turn act upon the NPR-11 receptor on AIA to modulate INS-1 peptide secretion. Closing the feedback loop, INS-1 acts on the AWC sensory neurons to modulate their responsiveness to sensory stimuli (Figure 1C). Although strong evidence from mutant and other studies demonstrate a functional NLP-1/NPR-11 relationship, other peptides have been shown to activate the receptor with EC_50_ values in the nanomolar range, including FLP-21 (1–10 nM) and FLP-18 [(SYFDEKK)SVPGVLRFa, 80–800 nM] (40). As mentioned above, the FLP-21 peptide modulates behavior in the context of food and other environmental parameters through activation of the receptor NPR-1, and is expressed in ADL, ASE, and ASH sensory neurons among others (61, 63, 111). ASE neurons in particular are implicated in food-dependent behavior, as they are mainly responsible for chemotaxis to water-soluble attractants (116, 124). Which neurons functionally act downstream of ASE in water-soluble chemotaxis has not been fully understood. AIA interneurons are prominent targets of ASE, hereby hinting on a functional FLP-21/NPR-11 interaction consistent with the observed *in vitro* data. Although this interaction has not been uncovered in previous studies, it remains interesting to investigate whether NPR-11 signaling in AIA by FLP sensory peptides can activate a neuropeptidergic feedback loop to modulate the gain or temporal properties of the sensory activation process, analogous to that for AWC olfactory neurons (118, 120). AIA could in that respect act as a local integrator of sensory information (Figure 1).

In addition to the regulation of foraging behavior and metabolism, feeding in *C. elegans* is closely linked to pharyngeal pumping activity (125). Pumping activity is regulated by an intrinsic pharyngeal nervous system (126), but neurohormones released from neurons extrinsic to this cellular system can also influence pumping behavior (127). Several FLPs act on pharyngeal muscle to either excite or inhibit pumping (34, 128, 129). Despite the disadvantage of its size, numerous electrophysiological studies have been performed to reveal the effect of FLPs on pharyngeal preparations in *C. elegans*. Surprisingly, many of the tested FLPs modulate action potential frequency, suggesting an impressive neurochemical complexity of the feeding circuit (34, 128, 129). Different FLPs have been found to exert opposite effects on action potential frequencies of pharyngeal muscles. Stimulatory peptides include FLPs derived from the *flp-5, 6, 8*, and *14* precursor genes, whereas others elicit inhibitory effects on serotonin-induced depolarization of pharyngeal muscles like *flp-1, 3, 9, 13*, and *16* encoded peptides. By using wild-type worms and mutants with deficits in synaptic signaling, it was shown that FLP-13 (APEASPFIRFa) acts directly on the pharyngeal muscle, while FLP-8 acts via the pharyngeal neuronal circuit (34). These results are consistent with the fact that the majority of excitatory and inhibitory peptides were encoded on genes shown to be expressed in the *C. elegans* pharyngeal nervous system (23). It therefore appears that multiple FLPs are involved in feeding behavior by modulating pharyngeal activity, as supported by findings in *A. suum* (54, 130–132). Using a modified pressure transducer, Brownlee and colleagues measured changes in intrapharyngeal pressure to monitor the contraction of the *Ascaris* radial pharynx muscle. PF3 (AF8, KSAYMRFa) causes a biphasic response in the pharynx of *A. suum*, with hyper-contraction following an initial relaxation. AF1 (KNEFIRFa), however, leaves the muscle in a more relaxed state (130, 132).

#### Effects of FLPs on locomotion

Besides locomotory activity, the control of the neuromuscular junctions that drive locomotion is certainly also to be considered in the context of nematode feeding, as it enables the worm to migrate toward food sources. Diverse inhibitory and excitatory activities have been reported in *A. suum* on body wall muscles upon the application of FLPs (54, 91, 133–136). Data obtained from this type of studies do, however, not always facilitate a better understanding of *in vivo* physiological functions. Although an array of FLP peptides clearly shows muscle-based effects, denervation of somatic muscle strips can alter or even abolish the activity of many FLPs indicating that FLP-receptors do not only reside on muscles (1, 92). Electrophysiological studies indeed demonstrate that FLPs, such as AF1, can act through modulation of neuronal conductance of motor neurons in addition to their muscle-based effects (137). Contrary to AF1, the effects of AF8 (KSAYMRFa) on somatic muscle of *A. suum* are uniquely differential and context-dependent. While application to dorsal muscles causes slow relaxation, AF8 has profound excitatory effects on ventral muscles (49). Remarkably, this is the only known nematode peptide to show such differential neuromusculatory activity. Stretton et al. have characterized the effects of *C. elegans* and *Ascaris* FLPs on the synaptic activity of *Ascaris* motor neurons (138). They identified five major neuronal response types, theoretically corresponding to at least five FLP-receptor subtypes. These differences might possibly be attributed to different receptors, second messengers, or the combination of both.

In order to understand the *in vivo* functions of neuropeptides, comprehensive analyses on locomotory behaviors of intact nematodes have been carried out. In *A. suum*, direct injection of synthetic FLPs into the body cavity elicits diverse behavioral responses including effects on body waveforms, body length, and paralysis (19, 96, 138, 139). Similarly, the normal locomotory behavior is severely disrupted when *flp*-coding genes are silenced by RNAi, as was shown for *flp-14* and *flp-32* in *G. pallida* (87, 140). FLPs also have profound impacts on the migrational abilities of parasitic nematodes toward their host, as illustrated by RNAi silencing of *flp-14* and *flp-18* in *M. incognita* (86, 141). Host delivered RNAi of *flps* as non-chemical based control strategy for parasitic nematodes is therefore gaining importance (142).

When on a solid surface, *C. elegans* lays on its side and moves in a sinusoidal fashion by undulating contractions and relaxations of dorsal and ventral longitudinal body wall muscles. These muscles use acetylcholine (ACh) and GABA as their primary excitatory and inhibitory neurotransmitters, respectively, and disruption of either of these transmitter biosynthetic pathways leads to severely uncoordinated locomotion (143–145). FLP-1 peptides are also required for the smooth sinusoidal movement of the animals, as inactivation of *flp-1* in *C. elegans* causes hyperactive movement (146). FLP-1 has been found to modulate ACh signaling (147), hereby providing a possible direct link to the regulation of locomotion. FLP-1 as well as FLP-18 peptides were also recently implied in the homeostatic balance of excitation-inhibition coupling in the locomotor circuit that drives body wall muscle contractions (148). This neuropeptide modulation primarily acts on the GABAergic neural transmission at the neuromuscular junctions, where FLP-18 peptides act directly on muscles via the NPR-5 receptor to either inhibit contraction or to promote relaxation. However, the FLPs also appear to have an effect on other cell types to coordinate locomotory output. In addition, Wani and co-workers performed a large-scale RNAi screen to identify genes that mediate endogenous dopamine signaling in *C. elegans*, an important system controlling worm locomotion (149). The identification of FLP-1 peptides in this study suggests that FLP signaling may be required for dopamine synthesis and release from dopaminergic neurons or for modulating dopamine signaling in dopamine-receptive neurons.

### FLP-coordinated regulation of feeding and nociception

One salient feature of neuropeptide modulation common to both vertebrates as invertebrates is their role in gating and controlling the gain of peripheral sensory inputs (150, 151). In vertebrates, FLP signaling has been repeatedly linked to the modulation of opioid signaling and nociception, whereas the opioid system participates in the regulation of feeding (11, 152, 153). This recurrent interplay makes it conceivable to state that FLP and opioid systems could interact to integrate feeding with stress. Such coordinated regulation would enable animals to decide whether to engage in feeding-related behaviors when presented with an attractive food source in the presence of aversive or noxious stimuli (11). Furthermore, the primary FMRFamide sequence is embedded within an endogenous mammalian opioid peptide derived from the Met-enkephalin precursor, suggesting that enkephalins and FLPs may have coevolved from a common ancestral peptide and share functional links (154). These findings imply that synergistic pathways between stress and feeding behavior might have been evolutionary conserved.

The coordinated regulation of food-dependent behavior (aggregation) and stress perception (nociception) has been thoroughly documented in *C. elegans*. The manifestation of aggregating behavior involves multiple pathways linking the RMG hub neuron by gap junctions to nociceptive (ASH and ADL), oxygen-sensing (URX), and chemosensory neuron spokes (98–100). Simultaneous ablation of ASH and ADL attenuates aggregation, implying that this behavior may be a response to repulsive or stressful environmental stimuli (98). Aggregation could supply a defense to the animal, with group feeding stimulating dauer formation or prompting the secretion of enzymes that inactivate bacterial toxins (98). The induction of solitary behavior by the FLP receptor NPR-1 hints that its actions may antagonize responses of ASH and ADL to stressful cues. As both neuron types synthesize FLP-21 (61), they are believed to be able to induce solitary behavior under certain conditions. Given that NPR-1 is expressed in ASH nociceptors, it may also directly modulate their sensory responses correlated to feeding state and food availability (99).

**Figure 2:**
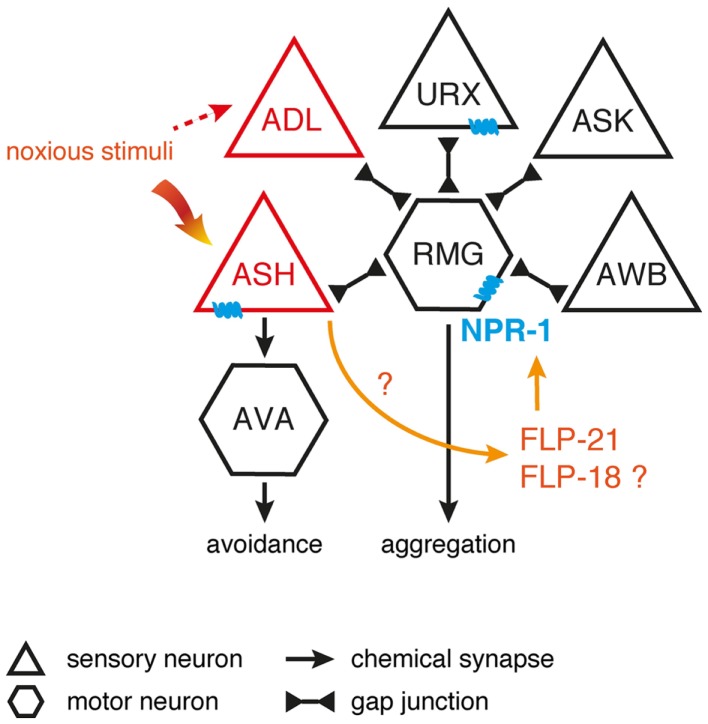
**Inhibition of the RMG interneuron by NPR-1**. Signaling from ASH and ADL neurons induces aggregation through gap junctions with RMG. RMG is the hub neuron of a gap junction network connecting various sensory neurons known to trigger aggregation. ASH and ADL also mediate acute avoidance behavior through synaptic signaling. Both types of connections are differentially regulated by the NPR-1 receptor, with FLP signaling inhibiting the gap junction driven activation of RMG and not being essential to ASH-mediated avoidance [adapted from Ref. (100,151)].

Surprisingly, NPR-1 is able to uncouple two overlapping circuits downstream of the ASH nociceptor (151). ASH utilizes glutamatergic synapses to signal to interneurons that control backward locomotion associated with the avoidance response to noxious stimuli (155, 156). In contrast, aggregation is driven by electrical gap junction signaling between ASH and the RMG hub neuron. Neuromodulation of RMG by NPR-1 uncouples the aggregation circuitry thus making it functionally silent, while sparing the function of the ASH-mediated avoidance circuit. This organization allows ASH to differentially generate behaviors depending on the neuromodulatory state, with aggregation occurring only when NPR-1 activity is low, and avoidance occurring regardless of modulation. One attractive hypothesis is that the dynamics of this circuit is differentially regulated by distinct sensory cues. A high intensity aversive cue might trigger the release of FLP-21 peptides and subsequently suppress the aggregation behavior, hereby facilitating efficient escape from highly noxious stimuli without the interference of motor programs for aggregation. The polymodal ASH nociceptor is exquisitely suited to detect various aversive stimuli, but other (FLP releasing) sensory neurons may also impinge on the RMG circuit. The RMG-hub-and-spoke circuit perfectly illustrates how information flow through worm circuits depends on neuromodulatory states defined by neuropeptides (Figure 2). The principle of circuit flexibility relying on connectivity modulation also extends to vertebrates as exemplified by stress-induced analgesia, an acute suppression of pain generated mediated by opioids (151, 157, 158).

### Nematode reproduction: FLP modulation of egg-laying and sexual behavior

FMRFamide-like peptide signaling modulates nematode reproductive behaviors such as egg-laying and copulation. Neuropeptides encoded by the *C. elegans flp-1* gene are suggested to modulate egg-laying rates, since *flp-1* deletion mutants show a defect in the timing of these events (159). This FLP-1-dependent regulation is furthermore dependent on food abundance (159). In a genome-wide RNAi study, Keating et al. (160) reported that knockdown of the FLP receptor FRPR-3 increases brood size and the rate of egg-laying (160). Besides genetic studies, FLPs have been directly tested for activity on muscles associated with the female reproductive system (134, 161). When applied to the ovijector of *A. suum*, for example, AF1 causes a biphasic effect transiently relaxing and then contracting the tissue, whereas both AF2 (KHEYLRFa) and PF3 (AF8, KSAYMRFa) have inhibitory effects (133).

**Figure 3:**
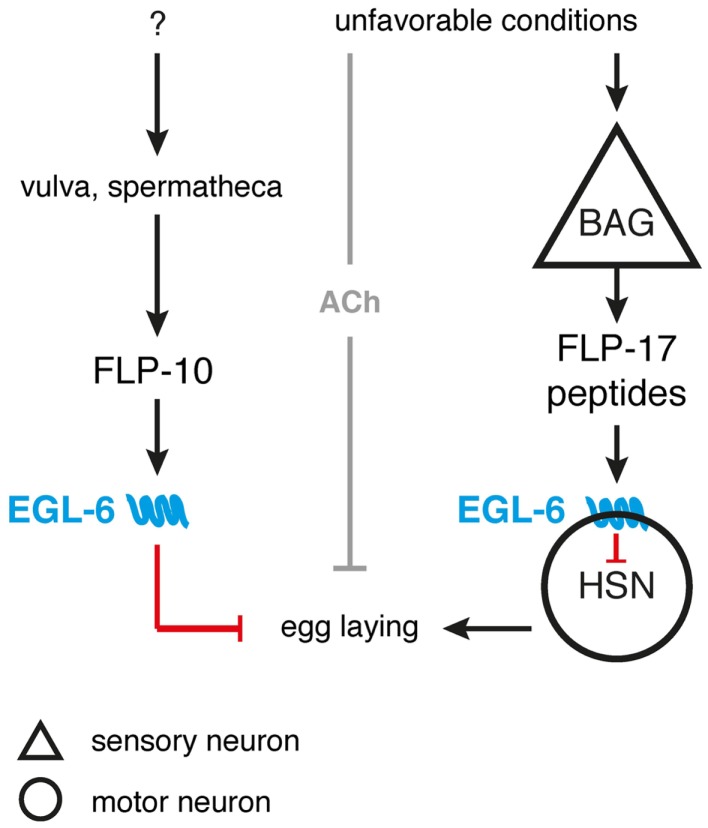
**FLP signaling suppresses egg-laying in unsuitable environments**. BAG neurons release FLP-17 neuropeptides in response to unfavorable conditions. These peptides are able to activate the EGL-6 receptor on HSN motor neurons, hereby inhibiting egg-laying. Release of FLP-10 by the vulva and spermatheca along with subsequent EGL-6 signaling further inhibits egg-laying, with the exact triggering stimuli still uncharacterized. Under unfavorable conditions, cholinergic signals (ACh) may be independently invoked by other sensory circuits to synergistically inhibit egg-laying [adapted from Ref. (53,81)].

Egg-laying in *C. elegans* is also modulated by *flp-10* and *flp-17* encoded peptides (53). These FLPs are able to activate the EGL-6 receptor that is present in the HSN motor neurons innervating the vulval musculature, hence regulating egg-laying behavior (53, 101, 162). In comparison to wild-type *C. elegans, egl-6* overexpression and gain-of-function mutants display slower egg-laying rates, suggesting an inhibitory receptor function (53). Both peptides encoded by *flp-17* are expressed in a pair of BAG sensory neurons, whereas *flp-10* is expressed in several neuronal and non-neuronal tissue. Laser-ablation and overexpression experiments suggest that the vulva and spermatheca are the principal source of the endogenous FLP-10 peptide acting on EGL-6 (23, 53). This leads to a simple model in which relevant sensory cues control FLP-10/FLP-17 secretion, hereby directly modulating the activity of the egg-laying motor neurons to suppress egg-laying in unsuitable environments (Figure 3). Inhibition of the HSN motor neuron by EGL-6 seems to be synergistically to cholinergic inhibition of egg-laying upon unfavorable conditions (53).

Although *C. elegans* populations almost entirely consist of self-fertilizing hermaphrodites, males arise infrequently under certain environmental conditions. Males strikingly differ from their hermaphrodite counterparts in their complex mating behavior in which males turn backwards along the hermaphrodite body until their tail contacts the vulva, after which copulation is engaged. Peptidergic signaling by FLP-8, FLP-10, FLP-12, and FLP-20 is required for the sensory transduction in male turning behavior (163). Loss-of-function mutations in corresponding genes each induce repeated turning, with males continually circling the hermaphrodite instead of initiating copulation after a single turn. Although these *flp-*genes are somewhat dispersedly expressed in various sensory neurons and interneurons, *flp-20* expression in the mechanosensitive cells completely rescues the mutant’s turning phenotype (163). FLP-20 is therefore hypothesized to convey somatosensory information to terminate the turning program and initiate copulation. How gender-specific modifications of the shared touch circuitry of male and hermaphrodite nervous systems contribute to copulatory behaviors still remains unknown.

### FLP signaling in learning behavior

A growing body of evidence, including from studies on mollusks and arthropods, implicates FLPs in the regulation of learning behavior (164–166). *C. elegans* displays a remarkable level of behavioral plasticity similar to that observed in higher organisms (95, 167, 168), including non-associative (adaptation, habituation) and associative learning behaviors (169). For example, *C. elegans* can learn to approach or avoid tastes, odors, oxygen, or temperatures that predict the presence or absence of food. Both short-term and long-term forms of memory have been demonstrated in *C. elegans* (95).

In *C. elegans*, FLP-20 is involved in tap habituation, a type of non-associative learning behavior (170). *C. elegans* reverses its locomotion in response to a non-localized mechanical stimulus generated by tapping the culture plate containing the animal, a behavior known as the tap withdrawal response. Repeated taps result in habituation as measured in a decrement of both the amplitude and the frequency of this reversal (171). Mutants for the *flp-20* gene show deficits in the relatively short-term 12-h memory following a massed training session. On the other hand, *flp-20* is not required for long-term memory of tap habituation that lasts up to 48-h after temporally spaced training in which the same amount of training is presented with interval resting periods (172). This and other studies illustrate how two types of memory within the same learning paradigm are induced by distinct molecular mechanisms that are differentially initiated depending on the temporal pattern of the training regimen. The *flp-20* gene is specifically required within the mechanosensory neurons that presumably release FLP-20 peptides to activate downstream neurons required for short-term memory consolidation. This type of memory correlates with a *flp-20*-dependent increase of synaptic vesicles in the terminals of the mechanosensory neurons (170). This and other studies suggest that the molecular changes underlying short-term memory arise and are maintained at the level of the sensory neurons. Pre-synaptic changes in particular seem indispensable, and likely entail differential release of signaling molecules to dampen the reversal response in the context of tap habituation.

## Conclusion

The nematode FLP system comprises an intertwined signaling network with a broad array of neuropeptides operating within an anatomically small nervous system. FLP diversity translates into a central role of this neuropeptide family in various aspects of nematode biology. Functional studies in nematodes support the evolutionary continuity of FLPs as key regulators of energy balance, feeding behavior, reproduction, and sensory modulation. In general, the FLP complement has shown to be widely conserved throughout the phylum though some peptides show a more restricted distribution, with the latter potentially as a consequence of adaptation to a specific lifestyle such as parasitism (2, 24, 42). The particular cellular distribution of FLPs appears not to be fully conserved across nematodes, in contrast to the slow rate at which the nematode nervous system evolves at the cellular level. Rapidly evolving peptide expression could therefore reveal to be an essential factor in the generation of species-specific behavior, furthermore facilitating the radiation of nematodes into a variety of habitats including as parasites of both animals and plants (12).

In *C. elegans*, the *flp-*genes have overlapping expression patterns, with at least half of all neurons expressing one or more FLPs (173). This implies that some neurons use a repertoire of FLP peptides in addition to other messengers, which may be deployed in a context-dependent way rendering these cells multifunctional. Such multiplexing could contribute to increase the complexity of information processing in a numerally simple nervous system, hereby supporting the rich behavioral palette of nematodes. Given their broad diversity and expression, neuropeptides are exquisitely suited to actively recruit particular cellular circuits depending on the environmental and internal context. This type of neuromodulation appears to be an irreducible part of circuit flexibility in the nematode nervous system (174).

The considerable amounts of data on nematode FLP function derived from neuronal and neuromuscular bioassays demonstrate an impressive complexity in the FLP signaling system. On the other hand, the knowledge of FLP-receptor interplay remains sparse, and most of our current understanding is derived from *C. elegans* in which several FLP-receptors have been coupled to their peptide ligands by *in vitro* assays and *in vivo* functional studies. A common theme in these studies is that a single receptor can be activated by multiple FLPs encoded by one or more genes. However, this apparent receptor promiscuity will need to be proven physiologically relevant, as a whole layer in the control of FLP signaling may reside in the spatiotemporal expression patterns of both receptor and ligand molecules. With the increasing number of completed genome projects and transcriptome resources, putative FLP-receptors can readily be identified using bioinformatics, and *C. elegans* data as a scaffold, broadening our view on FLP signaling in other nematodes. In addition, further receptor deorphanization and subsequent localization of these proteins will, together with the extensive data regarding FLP distribution, shed light on specific FLP functioning within the modulation and coordination of nematode behavior and physiology.

## Conflict of Interest Statement

The authors declare that the research was conducted in the absence of any commercial or financial relationships that could be construed as a potential conflict of interest.
