# Corrigendum: the FMRFamide-like peptide family in nematodes

**DOI:** 10.3389/fnins.2015.00120

**Published:** 2015-04-09

**Authors:** Katleen Peymen, Jan Watteyne, Lotte Frooninckx, Liliane Schoofs, Isabel Beets

**Affiliations:** Functional Genomics and Proteomics Group, Department of Biology, KU LeuvenLeuven, Belgium

**Keywords:** FMRFamide-like peptides (FLPs), nematodes, *C. elegans*, neuropeptide, G protein-coupled receptor, feeding behavior, reproduction

In Figure 1, NLP-1 neuropeptides (not NLP-11 as indicated in Figure 1) are released from AWC olfactory cells and activate the NPR-11 receptor. The AWC-released neuropeptide NLP-1 is stated correctly in the figure legend.

In Table 1, the activity of neuropeptides on *C. elegans* G protein-coupled receptors is indicated by an EC50 range or by an activity threshold when EC50 values could not be calculated. The activity thresholds are indicated in italic in the corrected Table 1 below.

**Table 1:** **Neuropeptide genes encoding FLPs in nematodes**.

***flp* gene^a^**	**Species^b^**	**C-terminal peptide consensus^c^**	***C. elegans* FLPs^d^**	***C. elegans flp* expression^e^**	***C. elegans* receptor interaction (EC_50_ range or *activity threshold*)^f^**	**References**
*flp-1*	*A. caninum, A. ceylanicum, A. suum, B. malayi, B. xylophilus, C. elegans, C. vulgaris, D. immitis, G. pallida, G. rostochiensis, H. concortus, H. schachtii, L. loa, M. arenaria, M. chitwoodi, M. hapla, M. incognita, M. javanica, M. paranaensis, N. brasiliensis, N. americanus, O. onchengi, O.volvulus, P.redivivus, P. trichosuri, S. ratti, S.stercoralis, T. muris, T. spiralis, W.bancrofti*	- [P/N/Q/A/] [N/T/D/S/K] [F/Y]LRFa	**SADPNFLRFa** **SQPNFLRFa** **ASGDPNFLRFa** **SDPNFLRFa** **AAADPNFLRFa** (K)PNFLRFa AGSDPNGLRFa ^*^(K)PNFMRYa	AIA, AIY, AVA, AVE, AVK, RIG, RMG, M5	NPR-22 (*100 nM*) NPR-4 (~0.4–9 μM) NPR-11 (~1–8 μM)	Geary et al., 1992; Rosoff et al., 1992, 1993; Schinkmann and Li, 1994; Nelson et al., 1998a; Kimber et al., 2001, 2002; Rogers et al., 2001; Lowery et al., 2003; Kim and Li, 2004; Husson et al., 2005, 2006; Kimber and Fleming, 2005; McVeigh et al., 2005; Mertens et al., 2006; Abad et al., 2008; Kikuchi et al., 2011; Jarecki et al., 2012; McCoy et al., 2014
*flp-2*	*A. caninum, A. suum, B. xylophilus, C. elegans, G. pallida, H. concortus, M. chitwoodi, M. hapla, M. incognita, M. javanica, N. americanus, N. brasiliensis, O.ostertagi, S. ratti*	[L/F/V/S/Q][P/R/M][G/R]EP[I/L]RFa	LRGEPIRFa **SPREPIRFa**	AIA, RID, PVW, I5, MC (ASI, M4, head muscles, an extra pair of cells in the head	FRPR-18 (~50 nM)	Nelson et al., 1998a; Kim and Li, 2004; McVeigh et al., 2005; Mertens et al., 2005; Kikuchi et al., 2011; Larsen et al., 2013; McCoy et al., 2014
*flp-3*	*A. suum, B. malayi, B. xylophilus, C. elegans, D. immitis, G. pallida, H. glycines, L. loa, M. arenaria, M. chitwoodi, M. hapla, M. incognita, O. volvulus, O. onchengi, S. ratti, W. bancrofti*	- [S/A/E/T/N][P/L][L/F/P]GTMRFa	SPLGTMRFa **TPLGTMRFa** **SAEPFGTMRFa** **NPENDTPFGTMRFa** **ASEDALFGTMRFa** **EDGNAPFGTMRFa** **EAEEPLGTMRFa** **SADDSAPFGTMRFa** NPLGTMRFa	IL1, PQR, SP, CP9	NPR-10 (~60–300 nM) NPR-4 (≥10 μM)	Nelson et al., 1998a; Rogers et al., 2001; Lowery et al., 2003; Kim and Li, 2004; Greenwood et al., 2005; Husson et al., 2005; McVeigh et al., 2005; Husson et al., 2007a; Abad et al., 2008; Li and Kim, 2010; Kikuchi et al., 2011; McCoy et al., 2014
*flp-4*	*A. caninum, A. ceylanicum, A. suum, B. malayi, B. xylophilus, C. elegans, D. immitis, H. glycines, N. brasiliensis, O. ochengi, O. volvulus, W. bancrofti*	- [A/T/G][Q/N/S/K][P/S][T/S]FIRFa	PTFIRFa ASPSFIRFa	ADL, ASEL, AVM, AWC, FLP, PHA, PHB, PVD, I5, I6, NSM	NPR-4 (~5–80 nM)	Nelson et al., 1998a; Lowery et al., 2003; Kim and Li, 2004; McVeigh et al., 2005; Li and Kim, 2010; Jarecki et al., 2011; Kikuchi et al., 2011; McCoy et al., 2014
*flp-5*	*A. caninum, A. suum, B. xylophilus, C. elegans, G. pallida, G. rostochiensis, H. concortus, H. glycines, M. arenaria, M. hapla, M. javanica, M. incognita, N. brasiliensis, N. americanus, P. penetrans, S. ratti*	- [G/A/N/K][A/Q/P]KFIRFa	APKFIRFa AGAKFIRFa **GAKFIRFa**	ASE, PVT, RMG, I4, M4, pharyngeal muscle, amphidial neuron (PB, I2), rays 1,5,7, HOB, P8	NPR-11 (~1–8 μM)	Nelson et al., 1998a; Rogers et al., 2001; Lowery et al., 2003; Kim and Li, 2004; Husson et al., 2005; McVeigh et al., 2005; Abad et al., 2008; Li and Kim, 2010; Kikuchi et al., 2011; McCoy et al., 2014
*flp-6*	*A. caninum, A. ceylanicum, A. suum, B. malayi, B. xylophilus, C. elegans, D. immitis, G. pallida, G. rostochiensis, H. concortus, H. glycines, L. loa, M. chitwoodi, M. hapla, M. incognita, M. paranaensis, N. brasiliensis, N. americanus, O. ochengi, O. ostertagi, O. volvulus, P. redivivus, S. ratti, S. stercoralis, T.circumcincta, W. bancrofti*	KS[A/S]YMRFa	**KSAYMRFa** (6x) ^*^pQQDSEVEREMM	ASE, AFD, ADF, ASG, PVT, I1 (one or two pairs of head cells), rays 2, 5, 6, 7		Maule et al., 1994a; Marks et al., 1998; Nelson et al., 1998a; Rogers et al., 2001; Kim and Li, 2004; Husson et al., 2005; McVeigh et al., 2005; Abad et al., 2008; Jarecki et al., 2010; Li and Kim, 2010; Kikuchi et al., 2011; McCoy et al., 2014
*flp-7*	*A. caninum, A. suum, B. xylophilus, C. elegans, G. pallida, G. rostochiensis, H. concortus, H. glycines, M. hapla, M. incognita, M. javanica, N. brasiliensis, O. ostertagi, S. ratti, S. stercoralis*	[A/T/S]P[F/L/M/I][D/Q/A/E]R[S/A/T] [S/A/T/K][M/L/I][A/V/I]RFa	**TPMQRSSMVRFa** (2x) **SPMQRSSMVRFa** (3x) SPMERSAMVRFa SPMDRSKMVRFa	ALA, AVG, PHB, PDA, PVW, RIC, SAA (RMDV/SMDV, PHA)	NPR-22 *(0.025–5 μM)* FRPR-3 (>1 μM)	Nelson et al., 1998a; Rogers et al., 2001; Mertens et al., 2004a; McVeigh et al., 2005; Husson et al., 2006; Mertens et al., 2006; Abad et al., 2008; Li and Kim, 2010; Kikuchi et al., 2011; McCoy et al., 2014
*flp-8*	*A. ceylanicum, A. suum, B. malayi, B. xylophilus, C. elegans, D. immitis, H. concortus, L. loa, N. americanus, N. brasiliensis, O. ochengi, O. volvulus, S. ratti, T. muris, T. spiralis, W. bancrofti, X. index*	KNEF[I/V]RFa	**KNEFIRFa** (3x)	AUA, PVM, URX (RMG,ADA, an extra pair of cells in the head), CP9		Cowden et al., 1989; Davis and Stretton, 1996; Nelson et al., 1998a; Rogers et al., 2001; Kim and Li, 2004; McVeigh et al., 2005; Yew et al., 2005; Li and Kim, 2010; Kikuchi et al., 2011; McCoy et al., 2014
*flp-9*	*A. caninum, A. ceylanicum, C. elegans, H. concortus, N. americanus, N. brasiliensis, O. ostertagi*	KPSFVRFa	**KPSFVRFa**		NPR-22 *(5 μM)*	Marks et al., 1999; Nelson et al., 1998a; Husson et al., 2005, 2006; McVeigh et al., 2005; Mertens et al., 2006; McCoy et al., 2014
*flp-10*	*A. ceylanicum, C. elegans, X. index*	- [A/T/M][R/A][S/G][G/S/K]Y[I/L]RFa	QPKARSGYIRFa	AIM, ASI, AUA, BAG, BDU, DVB, PQR, PVR, URX, vulD	EGL-6 (11 nM)	Nelson et al., 1998a; Kim and Li, 2004; McVeigh et al., 2005; Ringstad and Horvitz, 2008
*flp-11*	*A. suum, A. caninum, A. ceylanicum, B. malayi, B. xylophilus, C. elegans, D. immitis, G. pallida, G. rostochiensis, H. concortus, H. glycines, L. loa, M. hapla, M. incognita, M. paranaensis, N. americanus, N. brasiliensis, O. ochengi, O. ostertagi, O. volvulus, P. penetrans, R. similis, S. ratti, S. stercoralis, T. circumcincta, W. bancrofti*	- M/I/G/A/S][R/A][N/P][A/S/Q/E][P/L] VRFa	**AMRNALVRFa** **ASGGMRNALVRFa** **NGAPQPFVRFa** ^*^SPLDEEDFAPESPLQa	AUA, BAG, VD, DA, DD, DVB, LUA, PHC, PVC, SAB, URX, uvl, head muscle (socket cells), ray 4	NPR-22 *(0.75–2.5 μM)* FRPR-3 (~1 μM) NPR-4 (≥10 μM)	Nelson et al., 1998a; Lowery et al., 2003; Yew et al., 2003, 2005, 2007; Kim and Li, 2004; Mertens et al., 2004a; Husson et al., 2005, 2006; McVeigh et al., 2005; Mertens et al., 2006; Li and Kim, 2010; Kikuchi et al., 2011; McCoy et al., 2014
*flp-12*	*A. caninum, A. suum, B. malayi, B. xylophilus, C. elegans, D. immitis, G. pallida, G. rostochiensis, H. concortus, H. glycines, L. loa, M. arenaria, M. chitwoodi, M. hapla, M. incognita, M. javanica, M. minor, M. paranaensis, N. americanus, N. brasiliensis, O. ochengi, O. volvulus, S. ratti, W. bancrofti*	(K)[R/K/N]NKFEFIRFa	RNKFEFIRFa	AVA,AVJ, AVH, BAG, PDA, PVR, SAA, SDQ, SMB, (BDU), rays 1, 4, 5, 7, CP9		Davis and Stretton, 1996; Nelson et al., 1998a; Kimber et al., 2001, 2002; Kim and Li, 2004; Kimber and Fleming, 2005; McVeigh et al., 2005; Yew et al., 2005; Abad et al., 2008; Johnston et al., 2010; Kikuchi et al., 2011; McCoy et al., 2014
*flp-13*	*A. caninum, A. ceylanicum, A. suum, B. xylophilus, C. elegans, D. immitis, G. pallida, G. rostochiensis, H. concortus, H. glycines, L. loa, M. chitwoodi, M. hapla, M. incognita, M. javanica, N. americanus, N. brasiliensis, O. ochengi, O. ostertagi, O. volvulus, P. penetrans, P.pacificus, S. ratti, S. stercoralis, W. bancrofti*	-P[F/L/I][I/L/M/V]RFa	**AMDSPFIRFa** **AADGAPFIRFa** **APEASPFIRFa** (2x) **AADGAPLIRFa** **ASPSAPFIRFa** **SPSAVPIRFa** **SAAAPLIRFa** ASSAPFIRFa	ASE, ASG, ASK, BAG, DD, I5, M3, M5 (an extra pair of cells in the head), VSP	NPR-22 *(2.5–5 μM)*	Davis and Stretton, 1996; Marks et al., 1997, 2001; Nelson et al., 1998a; Kim and Li, 2004; Husson et al., 2005, 2006; Mertens et al., 2006; Abad et al., 2008; Jarecki et al., 2010; Li and Kim, 2010; Kikuchi et al., 2011; McCoy et al., 2014
*flp-14*	*A. caninum, A. ceylanicum, A. suum, B. malayi, B. xylophilus, C. elegans, D. immitis, G. pallida, G. rostochiensis, H. concortus, L. loa, M. arenaria, M. chitwoodi, M. hapla, M. incognita, M. javanica, M. paranaensis, N. americanus, N. brasiliensis, O. ochengi, O. volvulus, P. redivivus, P. trichosuri, P. penetrans, P.penetrans, R. similis, S. ratti, S. stercoralis, T. circumcincta, T. muris, T. spiralis, W. bancrofti*	KH[E/D][Y/F][L/V/I]RFa	**KHEYLRFa** (4x)		NPR-4 (≥10 μM) NPR-11 (~1–8 μM)	Cowden and Stretton, 1993; Maule et al., 1994b; Davis and Stretton, 1996; Li et al., 1999; Kimber et al., 2001, 2002; Rogers et al., 2001; Lowery et al., 2003; McVeigh et al., 2005; Kimber and Fleming, 2005; Yew et al., 2005; Husson et al., 2006; Abad et al., 2008; Jarecki et al., 2010; Johnston et al., 2010; Kikuchi et al., 2011; McCoy et al., 2014
*flp-15*	*A. ceylanicum, A. suum, C. elegans, H. concortus, N. americanus, N.braziliensis, O. ostertagi, T. circumcincta*	[R/D/G/A][G/V]P[T/S/Q]GPLRFa	**GGPQGPLRFa** **RGPSGPLRFa**	PHA, I2, socket/sheath cells (pharyngeal muscle, several cells in the head)	NPR-3 (~100–600 nM) NPR-4 (≥10 μM)	Li et al., 1999; Kubiak et al., 2003b; Lowery et al., 2003; Kim and Li, 2004; McVeigh et al., 2005; Mertens et al., 2006; Li and Kim, 2010; McCoy et al., 2014
*flp-16*	*A. caninum, A. ceylanicum, A. suum, B. malayi, B. xylophilus, C. elegans, D. immitis, G. pallida, G. rostochiensis, H. concortus, H. glycines, L. loa, M. hapla, M. incognita, N. americanus, N. brasiliensis, O. ochengi, O. volvulus, O. ostertagi, P. trichosuri, P. penetrans, P. vulnus, R. similis, S. ratti, W. bancrofti*	[A/G]QTFVRFa	**AQTFVRFa** (2x) **GQTFVRFa**			McVeigh et al., 2005; Abad et al., 2008; Li and Kim, 2010; Kikuchi et al., 2011; McCoy et al., 2014
*flp-17*	*A. caninum, A. suum, B. xylophilus, C. elegans, H. contortus, N. americanus, N. brasiliensis, O. ostertagi, S. ratti, S. stercoralis, X. index*	KS [A/S/Q][F/Y/L][V/I]RFa	KSAFVRFa (2x) KSQYIRFa	BAG, M5 (an extra pair of cells in the head), rays 1, 5, 7	EGL-6 (1–28 nM)	Li et al., 1999; Kim and Li, 2004; McVeigh et al., 2005; Ringstad and Horvitz, 2008; Li and Kim, 2010; Jarecki et al., 2011; Kikuchi et al., 2011; McCoy et al., 2014
*flp-18*	*A. caninum, A. ceylanicum, A. suum, B. xylophilus, C. elegans, D. immitis, G. pallida, G. rostochiensis, H. concortus, L. loa, M. chitwoodi, M. hapla, M. incognita, M. javanica, N. americanus, N. brasiliensis, O. ochengi, O. ostertagi, O. volvulus, P. pacificus, S. ratti, S. stercoralis, T. muris, T. spiralis, W. bancrofti*	- [P/Q/A][G/Q/D/A] [V/M/F/L][V/M/F/L]RFa	(DFD)GAMPGVLRFa EMPGVLRFa (SYFDEKK)SVPGVLRFa (3x) EIPGVLRFa SEVPGVLRFa DVPGVLRFa	AVA, AIY, RIG, RIM, M2 (M3, two extra pairs of cells in the head), rays 2, 6	NPR-4 (~5–80 nM) NPR-10 (~60 nM–4.6 μM) NPR-1 ((-32.2)–(-6.8))^**^ NPR-5a (~20–70 μM) NPR-5b (~30–800 nM) NPR-11 (~80 nM–8 μM)	Edison et al., 1997; Kimber et al., 2001; Lowery et al., 2003; Rogers et al., 2003; Husson et al., 2005, 2006; Kimber and Fleming, 2005; McVeigh et al., 2005; Yew et al., 2005; Abad et al., 2008; Cohen et al., 2009; Li and Kim, 2010; Kikuchi et al., 2011; McCoy et al., 2014
*flp-19*	*A. caninum, A. suum, B. malayi, B. xylophilus, C. elegans, D.immitis, G. pallida, H. concortus, H. glycines, L. loa, M. hapla, M. incognita, N. americanus, N. brasiliensis, O. ochengi, O. volvulus, P. penetrans, S. ratti, T. circumcincta, W. bancrofti*	- W[A/S][N/S/T][Q/K/S][V/L]RFa	**WANQVRFa** **ASWASSVRFa**	AIN, AWA, BAG, HSN, URX (an extra pair of cells in the tail), rays 5, 7, 9, CEM		Li et al., 1999; Rogers et al., 2001; Kim and Li, 2004; Husson et al., 2005, 2006; McVeigh et al., 2005; Abad et al., 2008; Li and Kim, 2010; Jarecki et al., 2011; Kikuchi et al., 2011; McCoy et al., 2014
*flp-20*	*A. suum, A. caninum, B. xylophilus, C. elegans, G. pallida, H. concortus, M. hapla, M. incognita, N. brasiliensis, P. trichosuri, S. ratti*	[A/V]MMRFa	AMMRFa (2x)	ALM, ASEL, AVM, LUA, PLM, PVC, PVM, PVR, RIB,AIB,(PVT)		Li et al., 1999; Kim and Li, 2004; McVeigh et al., 2005; Abad et al., 2008; Kikuchi et al., 2011; McCoy et al., 2014
*flp-21*	*A. caninum, A. suum, B. malayi, B. xylophilus, C. elegans, D. immitis, G. pallida, H. concortus, L. loa, M. hapla, M. incognita, N. americanus, N. brasiliensis, O. ochengi, O. ostertagi, O. volvulus, P. penetrans, P. pacificus, R. similis, S. ratti, S. stercoralis, T. circumcincta, W. bancrofti*	- [G/A/S/L][L/A]GPRPLRFa	GLGPRPLRFa	ADL, ASI, ASEASH, ASJ, ASK, FLP, URA, MC, M4, M2, SP, DVF, P6, P7, P9	NPR-1 (~2.5–100 nM) NPR-11 (~1–10 nM) NPR-5a (~0.6–5 μM) NPR-5b (~200–1500 nM)	Kubiak et al., 2003a; Lowery et al., 2003; Rogers et al., 2003; Greenwood et al., 2005; McVeigh et al., 2005; Abad et al., 2008; Cohen et al., 2009; Kikuchi et al., 2011; McCoy et al., 2014
*flp-22*	*A. caninum, A. ceylanicum, A. suum, B. malayi, B. xylophilus, C. elegans, D. immitis, G. pallida, G. rostochiensis, H. concortus, H. glycines, L. loa, M. hapla, M. incognita, N. brasiliensis, O. ochengi, O. ostertagi, O. volvulus, P. trichosuri, P. penetrans, P. pacificus, R. similis, S. ratti, S. stercoralis, T. circumcincta, W. bancrofti*	- [P/E/A/T/S][P/Q/G/E/N/S][S/G/V/A] KWMRFa	**SPSAKWMRFa** (3x)	AIM, ASG, AVA, AVG, AVL, CEP, PVD, PVW, RIC,AIZ, RIV, SMD, URA, uvl, 6 out of 9 CP	NPR-22 *(1 μM)*	Husson et al., 2005, 2006; McVeigh et al., 2005; Mertens et al., 2006; Abad et al., 2008; Kikuchi et al., 2011; McCoy et al., 2014
*flp-23*	*B. malayi, C. elegans, D. immitis, L. loa, O. ochengi, O. volvulus, T. circumcincta, W. bancrofti*	- [V/I/T][V/D/K][G/D/F][Q/G/F]QDFLRFa	VVGQQDFLRFa TKFQDFLRFa			Kim and Li, 2004; McVeigh et al., 2005; Li and Kim, 2010; McCoy et al., 2014
*flp-24*	*A. caninum, A. ceylanicum, A. suum, B. malayi, C. elegans, D. immitis, H. concortus, L. loa, N. americanus, O. ostertagi, O. ochengi, O. volvulus, S. ratti, W. bancrofti*	VP[S/N][A/P][G/A]DMM[V/I]RFa	**VPSAGDMMVRFa**			Kim and Li, 2004; McVeigh et al., 2005; Li and Kim, 2010; Jarecki et al., 2010; McCoy et al., 2014
*flp-25*	*A. caninum, A. suum, B. malayi, C. elegans, D. immitis, G. pallida, G. rostochiensis, H. concortus, L. loa, M. chitwoodi, M. hapla, M. incognita, M. javanica, N. americanus, N. brasiliensis, O. ochengi, O. volvulus, S. ratti, S. stercoralis, W. bancrofti*	- [D/A/S/N/T]YD[Y/F][V/I]RFa	DYDFVRFa **ASYDYIRFa**	ASE		McVeigh et al., 2005; Husson et al., 2006; Etchberger et al., 2007; Abad et al., 2008; Li and Kim, 2010; McCoy et al., 2014
*flp-26*	*A. caninum, A. ceylanicum, A. suum, C.elegans, N. americanus*	- [G/S][G/E][G/E/P][L/M/I][A/E]F[H/S/N] [P/A][N/D][D/M]L[A/S/T]LRFa	**(E)FNADDLTLRFa** **GGAGEPLAFSPDMLSLRFa** ^*^FRLPFQFFGANEDFNSGLT ^*^NYYESKPY			McVeigh et al., 2005; Husson et al., 2006; Li and Kim, 2010; McCoy et al., 2014
*flp-27*	*A. caninum, C. elegans, H. glycines, M. chitwoodi, M. hapla, M. incognita, M. javanica, M. paranaensis, N. americanus, R. similis*	[G/T/S/A][K/L/M]G[G/S]RMRFa	GLGGRMRFa ^*^pQPIDEERPIFME			McVeigh et al., 2005; Husson et al., 2006; Abad et al., 2008; Li and Kim, 2010; McCoy et al., 2014
*flp-28*	*A. suum, A. caninum, C. elegans, H. concortus, N. brasiliensis, O. ostertagi, P. penetrans, S. ratti*	- [V/I][L/F]MRFa	VLMRFa **APNRVLMRFa**			McVeigh et al., 2005; Husson et al., 2006; McCoy et al., 2014
*flp-31*	*B. xylophilus, G. pallida, M. chitwoodi, M. hapla, M. incognita, P. penetrans*	LYRPRGPPRFa				McVeigh et al., 2005; Abad et al., 2008; McCoy et al., 2014
*flp-32*	*A. caninum, B. xylophilus, C. elegans, G. pallida, H. concortus, M. hapla, M. incognita, N. brasiliensis, S. ratti*		AMRNSLVRFa			McVeigh et al., 2005; Abad et al., 2008; Li and Kim, 2010; Kikuchi et al., 2011; McCoy et al., 2014
*flp-33*	*A. suum, A. caninum, B. xylophilus, C. elegans, H. concortus, N. brasiliensis*		**APLEGFEDMSGFLRTIDGIQ** **KPRFa**			Husson et al., 2007b; Li and Kim, 2010; Kikuchi et al., 2011; McCoy et al., 2014
*flp-34*	*A. suum, A. caninum, B. malayi, B. xylophilus, C. elegans, D. immitis, G. pallida, H. concortus, M. hapla, M. incognita, L. loa, N. brasiliensis, O. onchengi, O. volvulus, W. bancrofti*		ALNRDSLVASLNNAERLRFa ^*^ADISTFASAINNAGRLRYa			Li and Kim, 2010; McCoy et al., 2014

a The flp-coding genes flp-29 and flp-30 were recently suggested to represent orthologues of C. elegans flp-28 and flp-2, respectively, and have been accordingly included in this table (McCoy et al., 2014).

b Species: Ascaris suum, Ancylostoma caninum, Ancylostoma ceylanicum, Brugia malayi, Bursaphelenchus xylophilus, Caenorhabditis elegans, Caenorhabditis vulgaris, Dirofilaria immitis, Globodera pallida, Globodera rostochiensis, Haemonchus concortus, Heterodera glycines, Heterodera schachtii, Loa loa, Meloidogyne arenaria, Meloidogyne incognita, Meloidogyne javanica, Meloidogyne hapla, Meloidogyne paranaensis, Necator americanus, Nippostrongylus braziliensis, Onchocerca ochengi, Onchocerca volvulus, Ostertagia ostertagi, Panagrellus redivivus, Parastrongyloides trichosuri, Pratylenchus penetrans, Pristionchus pacificus, Radolphus similis, Strongyloïdes ratti, Strongyloïdes stercoralis, Teladorsagia circumcincta, Trichinella spiralis, Trichuris muris, Wuchereria bancrofti, Xiphinema index.

c Sequences that start with a hyphen have variable N-terminal extensions.

d Peptides indicated in bold have been isolated from C. elegans. Peptides indicated with an asterisks are non-FLPs encoded by the indicated flp gene. The copy number of peptides encoded by the gene is indicated between brackets.

e Expression patterns were adapted from Li and Kim (2010), Etchberger et al. (2007) and Wormbase (www.wormbase.org).

f The approximate EC_50_ range for receptor activation is indicated between brackets and includes receptor activation by all peptides encoded by this precursor. Values indicated in italic are activity threshold values as EC_50_ values could not be calculated.

** Values represent alteration of current in response to neuropeptide application in Xenopus assay.

## Conflict of interest statement

The authors declare that the research was conducted in the absence of any commercial or financial relationships that could be construed as a potential conflict of interest.

